# Fragment-Resistant Property Optimization within Ballistic Inserts Obtained on the Basis of Para-Aramid Materials

**DOI:** 10.3390/ma15062314

**Published:** 2022-03-21

**Authors:** Katarzyna Kośla, Paweł Kubiak, Marcin Łandwijt, Wioleta Urbaniak, Agnieszka Kucharska-Jastrząbek

**Affiliations:** Institute of Security Technologies “MORATEX”, 90-505 Lodz, Poland; pkubiak@moratex.eu (P.K.); mlandwijt@moratex.eu (M.Ł.); wurbaniak@moratex.eu (W.U.); ajastrzabek@moratex.eu (A.K.-J.)

**Keywords:** armors and protection systems, body armors, para-aramid fibers, V50 ballistic performance, experimental ballistic techniques in protection, MCA analysis

## Abstract

A high protection level without an excessive weight is a basic assumption in the design of modern armors and protection systems. Optimizing armors is a task of development of the utmost importance, and is the subject of the work contained within this article. Optimization of ballistic inserts was carried out using multicriterial analysis (MCA), which enables the selection of the optimal composition, taking into account properties such as ballistic resistance, physicomechanical, and/or functional properties. For this purpose, various types of composite systems were produced and tested in terms of their fragment-resistant properties according to STANAG 2920 and the composite areal density of different ballistic inserts: Soft inserts made of Twaron^®^ para-aramid sheets, hard ballistic inserts made of multilayer hot-pressed preimpregnated sheets, and hybrid hard ballistic inserts prepared on the basis of multilayer hot-pressed preimpregnated sheets and ceramics. The application of MCA and performance of experimental fragment resistance tests for a wide spectrum of para-aramid inserts are part of the novelty of this work. The obtained test results showed that depending on the composition of the composite system, we could obtain a wide range of fragmentation resistance in the range of 300 to >1800 m/s, which depended on the areal density and type of composite system used. The results also confirmed that MCA is a good computational tool to select the optimal design of para-aramid ballistic inserts.

## 1. Introduction

Ballistic protection, especially hard and soft ballistic inserts, has undergone a significant revolution in recent years in aspects related to materials and design. Soft-body inserts consist of flexible ballistic materials. This type of armor is designed to protect against handguns and has a lower weight in comparison to hard-body inserts. For this reason, it is intended to be worn for an extended daily routine, for several hours. Soft inserts are typically constructed of multiple layers of ballistic-resistant materials [1,2,3]. The number of layers in soft ballistic inserts may impact their overall performance, which means the ability to absorb the energy of projectiles. In turn, hard inserts are made of rigid panels or plates. Hard ballistic armors may be constructed from compressed laminate sheets, ceramics, metals, or hybrid composites that incorporate more than one material [1,4,5,6,7]. Hard armors are designed to protect against greater threats (with a projectile velocity of more than 500 m/s) than soft armor [8]. They absorb and distribute the impact force through capture, deformation, and fragmentation of the bullet.

The present state of knowledge regarding ballistic armors is based on high-strength organic and inorganic materials. The fibers used in ballistics are characterized by a low density, a high tensile strength, and the ability to absorb high levels energy [9]. High-strength poly(phenylene-terephthalate-amide) fibers (Kevlar^®^, Twaron^®^), polyethylene fibers (HPPE) (Dyneema^®^, Spectra^®^), glass fibers (S-2 Glass), and products based on poly(p-phenylene-benzobisoxasole) (PBO) fibers (Zylon^®^) are commonly used [4,10,11,12]. Para-aramid fibers were first made in the 1960s by scientists from the DuPont company. The raw material that makes up the fibers is a long polyamide chain with at least an 85% share of amide groups (NHCO) connected directly with two benzene rings. Para-aramid fibers are molded from a liquid crystal polymer alloy, showing a high degree of molecular order. They were the first organic fibers with such a high tensile strength and modulus of elasticity [2]. These fibers were originally intended to replace steel in radial tires; however, they have been used with growing success in composites with special properties such as a high mechanical strength. The current shape of para-aramid fibers and their mechanical properties were achieved mainly by the specified chain structure. The fibers most often used in ballistic protective products are Kevlar^®^ para-aramid fibers produced by DuPont and Twaron^®^ produced by Teijin. These fibers are made from the mesomorphic phase solution of the polymer in concentrated sulfuric acid. Their tensile strength is five times greater than steel with the same weight, and the difference increases six times in water. This result is obtained due their low density [13]. Para-aramid fibers show good immunity to most chemicals, except for strong acids (e.g., formic acid and hydrochloric acid) and bases (e.g., sodium hydroxide and sodium hypochlorite). These fibers, with a low elongation at break and a high modulus of elasticity, are especially useful in areas that require high resistance to impact forces and abrasion, such as armor and personal protective equipment, cables, ropes, boat rigging, and parachutes. Unfortunately, most para-aramid fibers are sensitive to the effects of UV radiation, exposure to which results in visible changes to their natural color and decreases the long-lasting strength of the fibers [14]. Moreover, they are also characterized by high moisture absorption.

The para-aramids used in ballistic inserts usually have a woven structure, in which the weft and warp threads cross at right angles, forming a crisscross pattern. These yarns are continuous lengths of intertwined fibers with a significant ratio of length to their cross-section. This material is widely used in ballistic applications due to its impact resistance, lightweight nature, and high energy absorption ability—properties that make it especially useful for the production of body armor [15]. The possibilities of modifying the structure and assessing the properties of para-aramid fibers, woven and composites, in terms of their use in ballistic applications is still the subject of many studies. In research work performed by Roy et al. [16,17], the impact behavior of Kevlar-based composites (two-layered stitched para-aramid soft and stiff composites) was investigated. These composites were prepared using Kevlar^®^ 129 fabric as the reinforcement and epoxy resin or natural rubber latex (NR) as the matrix. It was observed that the stiff composites resulted in a 6.6 times higher puncture force, whereas for the flexible composites, the value was 2.8 times higher in comparison to the stitched panels. In terms of impact energy absorption, the stiff composites showed an 86% reduction in this value compared to the stitched panels, whereas for the flexible composites, a 73% increase was observed.

Khodadadi and coworkers [18] presented a comparison study of the behavior and energy absorption of Kevlar fabric composites and Kevlar impregnated with a polymer matrix under impact tests performed in a velocity range of 30–160 m/s. Thermoset (epoxy) and rubber matrices were used in order to study the effect of the matrix hardness, flexibility, and brittleness on energy absorption of the composite. The obtained results indicated that the ballistic performance of Kevlar–polymer composites depended on the used matrix. A rubber matrix enhanced the projectile energy absorption while maintaining the flexibility of the Kevlar–polymer composite. In view of this, better ballistic performance could be obtained via increasing the number of layers for the Kevlar–rubber composite. On the contrary, a thermoset (epoxy) matrix reduced the flexibility of the composite, and affected the deterioration of the ballistic properties as a result of restricting the fabric deformation. Another study showed the ballistic properties of aramid fabric with a graphene oxide coating. The use of graphene oxide in aramid fabric allowed for an increase in energy absorption by 50% compared to uncoated aramid fabric, which was probably related to the increased friction between the fibers. The main mechanisms of composite damage were microfibrillation failure, aramid rupture, and deformation and projectile fiber friction resulting from the cone formation in the underside of the composite [19]. These investigations showed that aramid fabric with a graphene oxide coating can be used for the production of materials for personal and automotive ballistic protection. A method of modifying para-aramid material with shear thickening fluids (STFs) and its enhancement of impact resistance was presented by Laha and Majumdar [20]. In this research, five weaved structures with varied thread densities of para-aramid yarns were prepared. The yarns were then modified with 60% shear thickening fluids to develop soft materials used in armors. As the results showed, modification upon using STFs improved the impact-resistance performance in the weaved structures, except for the plain weave with the highest thread density. The ascending order of weaves in terms of impact energy absorption before STF treatment was exactly the opposite of the ascending order of weaves after treatment with shear thickening fluid [20]. The ballistic characteristics of para-aramid woven fabrics and ultra-high molecular weight polyethylene unidirectional (UD) laminates were investigated by Yang and Chen [2]. The authors showed that with an increase in the total number of insert layers, the energy absorption of the Twaron^®^ para-aramid insert exhibited a downward trend, while that of the Dyneema^®^ UD unidirectional polyethylene laminate insert had a rising trend. They determined that in the case of para-aramid materials and polyethylene unidirectional laminates, the reverse trend in the ballistic performance was due to different types of damage. For the unidirectional polyethylene laminate, the dominant failure type was thermal damage, which could result in front layer performance degradation. Additionally, the unidirectional polyethylene laminate minimalized the back face signature (BFS) factor and showed a higher perforation ratio than the Twaron^®^ woven inserts. Based on the research, the authors designed an optimal hybrid insert composition by placing the Twaron^®^ para-aramid fabric before the Dyneema^®^ UD. This sequence of materials in the insert showed better ballistic parameters, such as a reduction in the perforation ratio and an improvement in the energy absorption compared to inserts with different arrangements. The obtained results indicated that material selection for hybrid designs should be based on their ballistic characteristics. 

Hybrid and homogenous packages based on Dyneema^®^ SB 71, Twaron^®^ UD 41, LFT-AT Flex, Felt no. 9, and Kevlar^®^ XP S307 were studied using a 7.62 × 25 mm Tokarev bullet. This research showed that hybrid packages with stiff antitrauma layers could reduce the BFS by approximately 10% compared to homogeneous inserts. The use of felt material decreased the BFS and increased the perforation resistance by stopping the projectile over a longer distance. However, when multiple stiff layers were used in a package, the perforation resistance was deteriorated by increasing the distance in which a projectile was stopped [15]. 

The main objective of the investigation presented in this paper was to choose a proper design and to optimize the material composition of para-aramid composites in terms of obtaining soft and hard ballistic inserts with the best physical and functional properties, especially the limit of ballistic protection (V50) and the areal density. For this purpose, various types of composite systems were produced and tested in terms of fragmentation-resistant properties:

- Soft ballistic inserts made of Twaron^®^ CT612 WRT and Twaron^®^ UD42 para-aramid sheets;
- Hard ballistic inserts made of:
  – Multilayer hot-pressed Twaron^®^ CT736 preimpregnated sheets;
  – Multilayer hot-pressed Twaron^®^ CT736 preimpregnated sheets and advanced ceramics based on Al_2_O_3_.

An novel element of this work was the performance of experimental fragment resistance tests for a wide spectrum of para-aramid inserts (both soft and hard inserts), as well as the use of MCA to optimize their construction.

The obtained test results showed that multicriteria analysis was a proper tool that enabled the selection of an insert in terms of the composite system’s composition and design, as well as the optimization of inserts, with the main factor being to ensure the best ballistic and functional properties. On the basis of this work, it was also determined that depending on the composition of the composite system, we could obtain a wide range of fragmentation resistance of 300 to over 1800 m/s, which depended on the areal density and type of composite system used. The results of part of a project that aimed to develop a next-generation explosive ordnance disposal (EOD) protective suit, designed to provide personal protection against the wave of overpressure, thermal radiation, and fragments generated by a bomb, are presented in this work. 

## 2. Materials and Methods

### 2.1. Materials

#### 2.1.1. Para-Aramid Materials

Three types of different para-aramid materials were used to produce the soft and hard ballistic inserts:

- Twaron^®^ CT612 WRT (Teijin Aramid GmbH, Wuppertal, Germany), which is a plain-woven para-aramid fabric with a linear density of its yarns of 550 and 500 dtex in the warp and weft directions, respectively.
- Twaron^®^ UD42 (Teijin Aramid GmbH, Wuppertal, Germany), which is a unidirectional (UD) laminate consisting of four para-aramid plies in a 0°/90°/0°/90° configuration and a polyethylene (PE) film that laminates the top and bottom layers of the para-aramid.
- Twaron^®^ CT736 (Teijin Aramid GmbH, Wuppertal, Germany), which is a para-aramid fabric impregnated with pure polyvinyl butyral (PVB) resin or PVB phenolic.

The properties of the Twaron^®^ para-aramid material are presented in Table 1 and Table 2.

#### 2.1.2. Ceramic Materials

To produce the ballistic inserts, hexagonal ceramics made from aluminum oxide (Al_2_O_3_ content 98%; CeramTec, Plochingen, Germany) were used. The technical parameters of the ceramic material are presented in Table 3.

#### 2.1.3. Tested Materials in the Form of Ballistic Inserts

The objects of the study were three types of ballistic inserts:

- Soft ballistic inserts made of Twaron^®^ CT612 WRT (Teijin Aramid GmbH, Wuppertal, Germany) or Twaron^®^ UD42 (Teijin Aramid GmbH, Wuppertal, Germany) para-aramid sheets, 250 mm × 250 mm in dimension and 3.6–10.0 mm in thickness in the case of Twaron^®^ CT612 WRT soft ballistic inserts, corresponding to the 21–62 para-aramid sheets and an areal density range of 2.5–7.5 kg/m^2^, or 2.2–6.8 mm in the case of Twaron^®^ UD42 soft ballistic inserts, corresponding to the 8–30 para-aramid sheets and an areal density range of 1.9–7.05 kg/m^2^.
- Hard ballistic inserts made of:
  – Multilayer hot-pressed Twaron^®^ CT736 (Teijin Aramid GmbH, Wuppertal, Germany) preimpregnated sheets—composite type 1, 250 mm × 250 mm in dimension and 3.0–12.5 mm in thickness, corresponding to an areal density of 9.2–21.4 kg/m^2^;
  – Multilayer hot-pressed Twaron^®^ CT736 (Teijin Aramid GmbH, Wuppertal, Germany) preimpregnated sheets and advanced ceramics based on aluminum trioxide (Al_2_O_3_) (CeramTec, Germany)—composite type 2, 250 mm × 250 mm in dimension and 7.0–16.5 mm in thickness, corresponding to an areal density of 23.0–34.2 kg/m^2^.

These were used in conjunction with soft ballistic inserts made of Twaron^®^ CT612 WRT (Teijin Aramid GmbH, Wuppertal, Germany) with an areal density of 5.0 ± 0.5 kg/m^2^.

#### 2.1.4. Preparation of Samples for Ballistic Tests

The soft ballistic inserts were prepared by cutting out and lagging (layering) the para-aramid material. The inserts were then sewn at the corners to prevent the layers from shifting relative to one another. The distance from the edge to the stitch was 2.0 ± 0.1 cm.

The hard ballistic inserts (composite type 1) were developed via the pressing process of para-aramid Twaron^®^ CT736 (Teijin Aramid GmbH, Wuppertal, Germany) sheets. The hot-pressing process was carried out at a temperature of 160–170 °C. A pressure of 4–30 MPa was used in the hot-pressing process. The pressing time and degassing process depended on the number of layers and the areal density in the package, and ranged from 2400 s for the additional ballistic inserts obtained from 10 layers of the preimpregnated sheets to 4500 s for the hard ballistic insert containing 35 layers of Twaron^®^ CT736 (Teijin Aramid GmbH, Wuppertal, Germany). The multilayer hot-pressed plate used in the construction of the hard ballistic inserts (composite type 2) had the same structure and areal density as for the type 1 hard ballistic inserts. The ballistic elements, including advanced ceramics (Al_2_O_3_) and the hot-pressed plate, were joined using silicone adhesive add-on Terostat MS 9399 (Henkel Poland, Warsaw, Poland).

The type 2 hard ballistic inserts were protected using suitable coatings. The hard ballistic inserts (types 1 and 2) were used in conjunction with soft ballistic inserts with an areal density of 5.0 ± 0.2 kg/m^2^, showing ballistic resistance (V50) equal to 620 ± 20 m/s according to STANAG 2920 methodology (NATO Standardization Office, Brussels, Belgium).

### 2.2. Testing Methods

#### 2.2.1. Assessment of the Physicomechanical Properties

Tests of the mechanical properties of the Twaron^®^ CT612 WRT (Teijin Aramid GmbH, Wuppertal, Germany) were performed according to the following standards: PN-ISO 3801:1977, PN-EN ISO 5084:1999, PN-EN ISO 13934-1:2013, PN-EN ISO 13937-2:2000, and PN-EN 1049-2:2000. The mechanical properties of the Twaron^®^ UD42 unidirectional laminate and Twaron^®^ CT736 preimpregnated sheets were tested according to the standards PN-EN ISO 2286-2:2016, PN-EN ISO 2286-3:2016, and PN-EN ISO 1421:2001.

Determination of the angle of bending stiffness for the flat textile products was achieved using the constant sample angle method according to the PN-73/P-04631:1974 standard. Tests were conducted for a single sheet of para-aramid Twaron^®^ CT612 WRT (Teijin Aramid GmbH, Wuppertal, Germany) and Twaron^®^ UD42 (Teijin Aramid GmbH, Wuppertal, Germany) materials. In order to determine the parameters, 10 samples with dimensions of 300 mm × 30 mm were cut from the section of the product to be tested in the longitudinal direction, and 10 in the transverse direction. Then, the test samples were placed on the horizontal plane of the measuring instrument and loaded with a metal gauge. The gauge with the sample was moved at a speed of 1 cm/s. The length of the hanging part of the package was determined on the measuring scale with an accuracy of 1 mm. The average length of the overhang was calculated as the arithmetic mean of all measurements for both sides of the package. The bending length (c—the ability of the product to deflect under its own weight, resulting from the product stiffness and unit weight) was determined as:

c = L/2,(1)

where L is the average length of the overhang—the length of the horizontally extended sample at which it will meet, under its own weight, with the BC plane inclined horizontally at an angle of 41°30′ (cm).

The unit bending stiffness (G) refers to the resistance of a body with a unit width against deformations caused by the action of external bending forces. The bending stiffness, expressed in millinewton meters, is numerically equal to the amount of bending moment needed to change the curvature by 1 cm^−1^ of the sample width, calculated using the formula:

G = 10^−6^ × m_F_ × c^3^ × g, (2)

where m_F_ is the areal density (kg/m^2^), c is the bending length (cm), and g is the Earth’s gravity (9806 m/s^2^).

The bending modulus (q) is the ratio of bending stiffness to the moment of inertia of the sample’s cross-section over the neutral axis, characterizing the stiffness of the sample material regardless of its dimensions, and is expressed as:

Q = (1.2 × 10^4^ × G)/a, (3)

where a is the average thickness (mm).

The overall bending stiffness factor (Go) was defined as follows:

Go = √G_w_ × G_p_, (4)

where G_w_ is the bending stiffness in the longitudinal direction, and G_p_ is the bending stiffness in the transverse direction.

The areal density was determined using the following equation:

D = (m × 10^6^)/A (5)

where m is the weight of the sample (g) and A is the surface area of the sample (mm^2^).

The thickness of the ballistic inserts was defined as the distance between the opposite surfaces of the sample. A thickness test was carried out by placing a sample between a pressure foot and a thickness gauge table. The pressure foot diameter used for measurements was 9 mm, while the table diameter was 50 mm. The sample positioning time was equal to 10 s. During the measurements, a preliminary pressure of 2.0 ± 0.2 kPa was used.

#### 2.2.2. Fragmentation Resistance Test

The samples intended for the assessment of fragmentation resistance were types 1 and 2 soft and hard ballistic inserts with dimensions of 250 mm × 250 mm, used together with “soft” ballistic inserts with an areal density of 5.0 ± 0.5 kg/m^2^. To determine the fragmentation resistance, STANAG 2920 was used. The test was performed in a “dry” state at a temperature of 20 ± 5 °C and a relative air humidity of 65 ± 10%. At least six shots made with a FSP.22 steel fragment with a hardness of 27 ± 3 HRC, a mass of 1.10 ± 0.03 g, a diameter of 5.46 ± 0.05 mm, and a length of 6.35 mm were fired for each sample. Half of the fired shots caused partial perforation of the insert, and the other half caused total perforation. Fragmentation resistance was determined by the limit of ballistic protection, V50, defined as the velocity at which, using the named projectile and target material, the estimated probability of perforation was 0.5 within a velocity spread of ∆ ≤ 40 m/s.

#### 2.2.3. Multicriterial Analysis (MCA)

Selection of the configuration for the soft and hard ballistic inserts obtained on the basis of para-aramid materials was performed on using the multicriterial analysis (MCA) results. In this analysis, the general coefficient of quality (GSQ) and the general quality class (GQC) were determined in order to select the optimal variants of the soft and hard ballistic inserts. MCA was performed according to methodology described in [21,22,23,24].

The following parameters (aggregated into two groups) were applied in the MCA:

(1) Physical properties (areal density and thickness);
(2) Functional properties (V50 ballistic limit for FSP.22 fragments and price of materials used for the ballistic insert preparation).

In this study, the validities presented in Table 4 were used for MCA realization.

The general coefficient of quality (GSQ), quality class, and sectional coefficient of quality (SCQ) were determined in accordance with the methodology presented by Struszczyk et al. [21] and Zurek et al. [24]. Generally, the GSQ and SCQ ranges are estimated to be between 0 and 1, where 0 is the worst quality and 1 is the ideal quality. The quality coefficients were classified into an appropriate quality class (GQC), where 0 indicated the ideal, and 9 was the most unfavorable [21,24].

## 3. Results and Discussion

### 3.1. Fragment-Resistant Property Optimization of Para-Aramid Soft Ballistic Inserts

Optimization of the ballistic properties of the para-aramid soft ballistic inserts was performed by means of MCA. Using a computational tool in the form of MCA, the SCQ and C coefficients for the physical and functional property groups were determined. MCA was also used to specify the values of the general coefficient of quality and the general quality class for ballistic liners made of para-aramid Twaron^®^ CT612 and Twaron^®^ UD42 materials. The results of the MCA are summarized and presented in Table 5 and Table 6.

On the basis of the results of the MCA (Table 5 and Table 6), it was determined that the optimum variants of the soft ballistic inlays based on Twaron^®^ CT612 were to be designated as CT612_1 and CT612_2. These variants had a GSQ within the range of 0.55–0.61 and a GQC factor equal to 4. For the ballistic insert obtained from Twaron^®^ UD42, the MCA selected UD42_1 and UD42_3. In this case, the GSQ factors were 0.61 and 0.63, respectively, and the GQC was equal to 4. The above soft ballistic inserts had the most optimal ratio of physical properties (surface mass and thickness) to functional properties (resistance to FSP.22 and the estimated price of raw materials needed to make the proper ballistic insert). According to the MCA, the least-effective designs were CT612_5, UD42_2, and UD42_5, for which an increase in the surface mass and thickness did not result in a significant increase in fragmentation-resistance properties.

**Figure 1:**
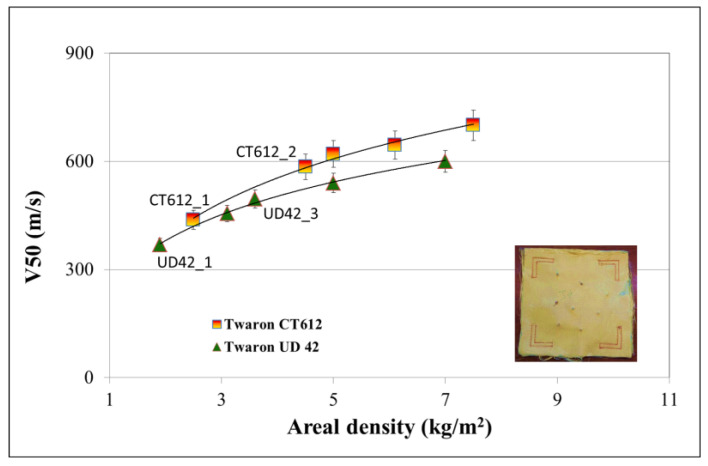
Soft ballistic inserts made from Twaron^®^ CT612 and Twaron^®^ UD42 after FSP.22 fragment resistance tests.

Additionally, the results of the dependence of the V50 value for the FSP.22 fragment on the areal density of the two selected types of soft ballistic inserts, where one was made with fabrics based on para-aramid fibers (Twaron^®^ CT612) and the other was made with unidirectional laminate (Twaron^®^ UD42), are presented in Figure 1.

Slightly lower V50 values were obtained for the soft ballistic inserts based on Twaron^®^ UD42 compared to the inserts made of Twaron^®^ CT612 in the entire tested area weight range of 2 to 7 kg/m^2^. With an increase in the number of layers of para-aramid sheets, and thus the areal density of the soft ballistic insert, the value of the V50 ballistic protection limit increased. In the case of the CT612 inserts, the V50 values obtained ranged from 440 to 700 m/s, and for the inserts obtained from the unidirectional laminate Twaron^®^ UD42, the values ranged from 370 to 600 m/s. The mechanisms discussed below influenced the differences in the obtained V50 values.

**Figure 2:**
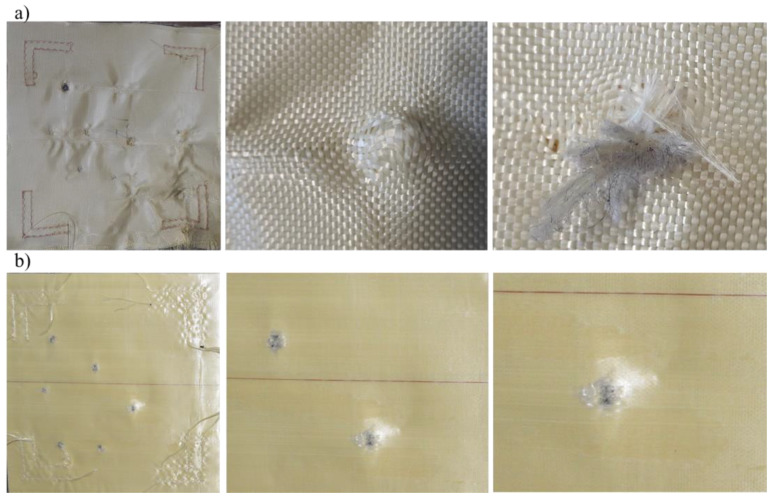
Soft ballistic inserts after FSP.22 fragment resistance tests: made from Twaron^®^ CT612 (**a**) and Twaron^®^ UD42 (**b**).

The mechanism of net ballistic debris retention for the woven fabrics, as well as their greater elasticity/deformability, withstood greater impact forces, while the structure of the nonwoven material deteriorated at lower shard impact values. In addition, the visual assessment of the soft ballistic inserts carried out after the ballistic tests indicated that stopping of the fragment and absorption of the impact energy occurred as a result of yarn rupture, yarn extension, and yarn pull-out (Figure 2a). These observations were in line with the research performed by Majumdar and Laha [25], who also reported these three major modes of energy absorption during impact: yarn stretching, pull-out of the fabric mesh that occurs in the penetration of hemispherical projectiles, and breakage.

Additionally, Nilkantan et al. [26] showed that yarn pull-out or slippage is the major mode of energy absorption. On the contrary, Hwang et al. [27] concluded that energy absorption is largely dependent on the rupture of primary yarns, the failure of secondary yarns, yarn pull-out, and fibrillation. On this basis, it can be concluded that one of the major causes behind the energy absorption is yarn pull-out, which is a function of inter-yarn friction [16]. Figure 2b shows the back face of a soft ballistic insert established on the basis of unidirectional laminate Twaron^®^ UD42. It is possible to observe the pushing out of the laminate and the occurrence of cones formed from the material, fiber breakage, PE, and fiber–matrix debonding; however, no pull-out of the para-aramid fibers was observed. The reason for such changes in the structure of the material as a result of the impact of high-energy shocks was a faster propagation of longitudinal wave during impact.

These observations were consistent with the literature data obtained for ballistic armor on the basis of K-Flex UD nonwoven fabrics [28] and ultra-high molecular weight polyethylene (e.g., Spectra Shield^®^ LCR) [29]. Moreover, Chocron et al. [30] and Yuan [31], in their works, did not observe fiber stretching or fibrillation, but they reported plugging and bulging deformation on the back face of inserts.

The dependence of the V50 value on the area weight in both cases was not linear, and in the range of area densities above 5 kg/m^2^, the differences in the values of the limit of the ballistic protection were only between 10% and 12%. The obtained values led to the conclusion that for lower velocities of 0.22 caliber fragments, soft ballistic inserts made with woven or unidirectional laminates of Twaron^®^ CT612 or UD42 para-aramids worked well. After exceeding a certain speed limit value of the impact of the fragment, increasing the number of layers of the para-aramid material did not result in significant changes, and more efficient material solutions should be used. Therefore, in Section 3.2, the results of research related to the assessment of the properties of fragmentation-resistant hard ballistic inserts are presented.

### 3.2. Optimization of the Fragment-Resistant Properties of Para-Aramid and Ceramic Para-Aramid Hard Ballistic Inserts

In this subsection, the determined V50 values are given for the following models of hard ballistic inserts connected with soft ballistic inserts of an areal density of 5.0 ± 0.5 kg/m^2^ containing Twaron^®^ CT612 in their structure:

- Composite type 1 (C1)—multilayer hot-pressed Twaron^®^ CT736 preimpregnated sheets;
- Composite type 2 (C2)—multilayer hot-pressed Twaron^®^ CT736 preimpregnated sheets and advanced ceramics based on aluminum trioxide (Al_2_O_3_) with a thickness of 3.50 ± 0.02 mm.

The tested hard ballistic inserts differed in their areal densities. Optimization of the fragment-resistant properties of these hard ballistic inserts was performed using MCA, in accordance with the data presented in Table 7 and Table 8.

The results collected using multicriteria analysis showed that the optimal variants of the composite system type 1 were C1_1–C1_3 (specified in Table 7 and Table 8), for which the coefficient GSQ was in the range of 0.56–0.63 and the GQC factor was 4. In the case of the composite system type 2, the realized MCA selected the C2_2 configuration, for which the GSQ coefficient was equal to 0.65 and the GQC was 3. The above-indicated composite systems had the most optimal ratios of the indicated physical and functional properties.

**Figure 3:**
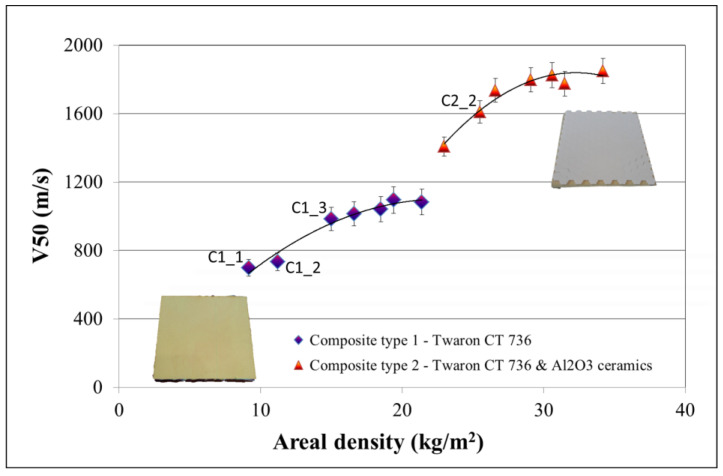
V50 ballistic protection limit by areal density of the tested composite system obtained for composites type 1 and 2.

Additionally, the differences between the V50 ballistic limit parameter and areal density of the individual ballistic inserts (composite types 1 and 2) are summarized in Figure 3.

The para-aramid ballistic inserts obtained as a result of heat-pressure pressing of Twaron^®^ CT736 showed resistance to the FSP.22 type fragment in the range of 700–1100 m/s depending on the mass per unit area (9.0–21.5 kg/m^2^). Initially, an increase in the number of layers of para-aramid material, and thus the areal density of the composite (areal density in the range of 9–16.5 kg/m^2^), resulted in a significant increase in the resistance to the FSP.22 fragment of the tested system (V50 values of approximately 700 to over 1000 m/s were obtained). However, increasing the areal density of the para-aramid material above 16.5 kg/m^2^ did not result in a significant increase in the limit of ballistic protection. The alterations between the V50 values obtained for the inserts with areal densities in the range of 18.5–21.5 kg/m^2^ and the input with an areal density of 16.5 kg/m^2^ were only approximately 60 m/s. Thus, an increase in the area weight of the pressed para-aramid composite obtained from Twaron^®^ CT736 by 23% above the value of 16.5 kg/m^2^ resulted in a change in the obtained V50 values of only 5–6% in relation to a V50 value equal to 1100 ± 15 m/s.

A similar relationship was observed for inserts obtained by combining pressed para-aramid plates and hexagonal Al_2_O_3_ ceramic elements used together with soft ballistic inserts with a areal density of 5.0 ± 0.5 kg/m^2^ containing Twaron^®^ CT612 in their structure. The differences in the ballistic protection limit for hybrid ballistic inserts made of a combination of pressed para-aramid plates and hexagonal Al_2_O_3_ ceramic elements with areal densities of 29 and 34 kg/m^2^ were less than 3% compared to the lighter versions of the composite (with an area weight of 26.5 kg/m^2^ containing fewer layers of pressed Twaron^®^ CT736), and were within the measurement error.

**Figure 4:**
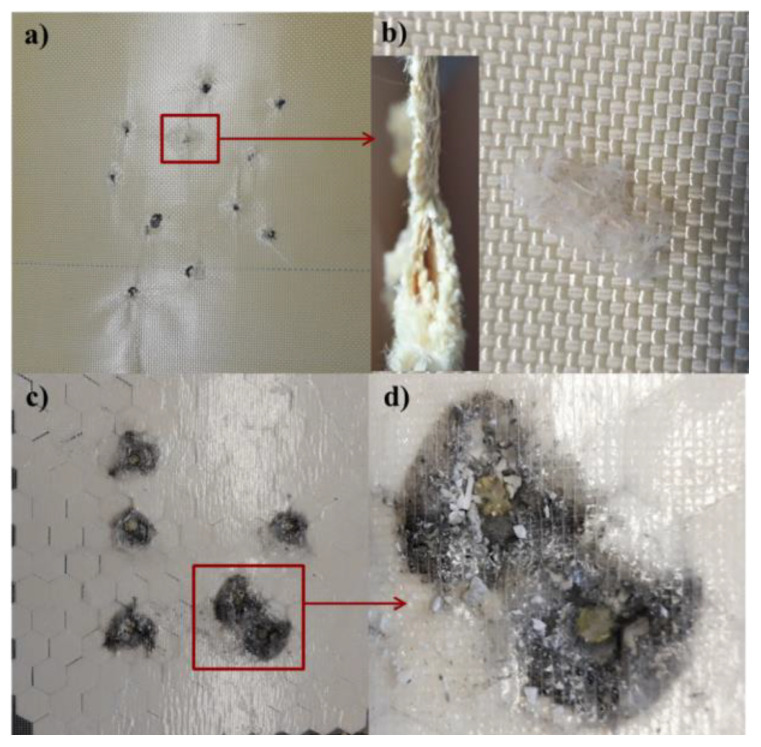
Hard ballistic inserts: Composite type 1—multilayer hot-pressed Twaron^®^ CT736 preimpregnated sheets (shooting side) (**a**); composite type 1—visible delamination and para-aramid yarn breakage (outlet side of fragment) (**b**); composite type 2—multilayer hot-pressed Twaron^®^ CT736 preimpregnated sheets and Al_2_O_3_ (shooting side) (**c**); composite type 2—ceramic elements cracked as a result of the impact of the FSP.22 fragment (**d**).

On the basis of the obtained results presented in Figure 4, it can be determined that the increase in the resistance to the FSP.22 type of fragmentation for the additional ballistic inserts obtained as a result of heat-pressure pressing of the Twaron^®^ CT736 para-aramid material, as well as hybrid inserts containing ceramic elements, was not linear. As the areal density increased, the V50 value increased; however, after exceeding a certain areal density limit value, the obtained values of the V50 ballistic protection limit remained at a comparable, almost unchanged level. This was a consequence of the mechanisms responsible for the ballistic properties and characteristics of a given type of composite.

As indicated by the literature data, a resin coating in ballistic composites increases the bending resistance of the fabric and enhances the resin/fabric insert’s resistance to inward deformation, thereby improving the ballistic performance [8,32]. Khodadadi et al. [18] showed that due to the brittleness of the thermosetting material, the damage to the matrix occurred around the bullet impact; however, it did not always lead to perforation of the composite. In their work, Khodadadi et al. [18] also pointed out that delamination was the second critical damage mode under high-velocity impact and was produced by interlaminar stress; while fiber breakage was the third mechanism presented, especially in rubber matrix composites. Convergent views were presented by Clifton and coworkers [33].

In the case of hard ballistic inserts obtained on the basis of Twaron^®^ CT736, the mechanism responsible for the ballistic properties of the tested system also include yarn breakage and delamination (Figure 4a,b). These mechanisms made it possible to stop the FSP.22 fragment acting on the composite at speeds of up to 1100 m/s. This was a significant fragment speed, considering the data presented by Colakoglu et al. [34], who showed that the V50 ballistic limit of 20 layers of Kevlar-29-reinforced PVB resin composites was 680 m/s, whereas Kevlar 29 without resin impregnation could only achieve 500 m/s. On the basis of the presented data, it also was determined that for a release velocity of the FSP.22 fragment of higher than 1100 m/s, these mechanisms would not be sufficient to stop it.

Therefore, a composite of a different structure should be used, such as hybrid composites consisting of a pressed para-aramid plate and ceramic elements. In this type of hard ballistic insert, the brittle fracture mechanisms of ceramic elements determine the ballistic resistance of the hybrid composite system and are the main component of resistance at fragment-to-composite interaction velocities higher than 1100 m/s (Figure 4c,d). The mechanisms of the projectile’s impact on ceramic ballistic inserts described in the literature indicated that the basic role of ceramics is to blunt the tip of the projectile, break it into fragments, and absorb some of its energy through brittle fracture of the ceramic elements of the ballistic composite. It was also determined that the role of a composite made of compressed polyethylene or para-aramid layers, to which a ceramic layer is attached, is to retain the projectile core fragments through elastic deformation and absorption of kinetic energy [35]. Energy absorption occurs through a combination of deformation, fiber pull-out, and delamination of the composite [36].

The destruction mechanism of ceramic elements was also the subject of research by Fejdys et al. [5], Cegla [35], Hogan et al. [37], Magier [38], and Reddy et al. [39]. Based on the situation observed in Figure 4c,d, it can be determined that the ceramic layer of the hard ballistic insert (composite system 2) played an analogous role, as indicated in the above literature data.

## 4. Conclusions

Based on the results collected in this research, it was determined that with the applied adaptation of multicriterial analysis, it was possible to choose the best-quality variants of ballistic inserts. MCA could also be helpful to describe the mechanism of the fragment-resistant behavior in terms of the physical properties of inserts with various configurations. Using MCA, the grouped parameters describing the performance and safety of the para-aramid soft and/or hard ballistic insert variants were converted to criterial markers.

Concluding the performed research, for lower velocities (400–700 m/s) of 0.22 caliber fragments, soft ballistic inserts made from para-aramid woven or unidirectional laminates work well. The mechanisms were related to rupture, extension, and pull-out of para-aramid yarn in the case of using woven materials; in the case of using unidirectional laminates, the mechanisms were related to pushing out and formation of a cone by the laminate, as well as para-aramid fiber breakage and separation, and delamination of the fibers from the polyethylene film were responsible for stopping the fragments. It was estimated that after exceeding a certain limit value of the fragment speed and its impact on the ballistic composite (>700 m/s in the research presented in this article), these mechanisms were not sufficient to stop the penetrator, and even increasing the number of para-aramid material layers did not result in significant changes or increases in the V50 parameter. However, this thesis should be supported by additional research.

In order to increase the ballistic protection limit, the structure of the applied ballistic system should be changed, and a combination of soft and hard ballistic inserts made of para-aramid plates obtained by thermal pressure pressing processes or hybrid inserts obtained by combining pressed plates and ceramic elements should be used. For FSP.22 fragments with speeds of 700–1100 m/s, composite inlays obtained from a combination of pressed plates with a soft insert are suitable, for which the ballistic properties depend on the matrix delamination and damage and yarn-breakage mechanisms. On the contrary, for V50 in the range of 1100–1800 m/s, the pressed plates should be replaced with hybrid inserts, including a combination of pressed para-aramid plates and ceramic elements, for which the ballistic properties are determined by the processes of brittle fracture of ceramics.

## Figures and Tables

**Table 1:** Physicomechanical properties of Twaron^®^ para-aramids (Teijin Aramid GmbH, Wuppertal, Germany).

Parameter	Unit	Type/Value	Test Method	Type/Value		Test Method
Material	-	Twaron^®^ CT612 WRT	Manufacturer’s declaration	Twaron^®^ UD42	Twaron^®^ CT736 (coated on one side)	Manufacturer’s declaration
Areal density	g/m^2^	123 ± 2	PN-ISO 3801:1993	238 ± 11	463 ± 3	PN-EN ISO 2286-2:1999
Thickness	mm	0.17 ± 0.03	PN-EN ISO 5084:1999	0.27 ± 0.02	0.57 ± 0.02	PN-EN ISO 2286-3:2000
Maximum tensile force	N		PN-EN ISO 13934-1:2013			PN-EN ISO 1421:2001
—Warp		5700 ± 250		7700 ± 280	17,400 ± 800	
—Weft		5800 ± 300		5100 ± 280	16,500 ± 200	
Elongation at break	%					
—Warp		5.0		5.8	6.6	
—Weft		6.5		3.0	7.8	
Tear strength	N	It does not tear	PN-EN ISO 13937-2:2002	It does not tear		PN-EN ISO 4674-1:2005
Ends Picks	Per 10 cm	112 ± 2 108 ± 2	PN-EN 1049-2:2000	N/A *	127 ± 4 127 ± 4	PN-EN 1049-2:2000

* N/A—impossible to test due to double-sided (top and the bottom) layers of lamination.

**Table 2:** Bending stiffness for single Twaron^®^ layers determined in accordance with the PN-73/P-04631:1974 standard.

Parameter/Material	Unit	Twaron^®^ CT612 WRT	Twaron^®^ UD42
**Longitudinal direction**			
Average of overhang length	(cm)	16.3	22.0
Unit bending stiffness, G_w_	(mNm)	0.623	3.280
Bending modulus, q_w_	(10^4^ kPa)	3.56	19.68
Bending length, c_w_	(cm)	8.0	11.0
**Transverse direction**			
Average of overhang length	(cm)	13.9	21.6
Unit bending stiffness, G_p_	(mNm)	0.417	3.100
Bending modulus, q_p_	(10^4^ kPa)	2.39	18.6
Bending length, c_p_	(cm)	7.0	10.8
General unit bending stiffness, G_o_	(mNm)	0.529	3.190

**Table 3:** Physical and mechanical parameters of the Al_2_O_3_ ceramics (CeramTec, Plochingen, Germany).

Parameter	Density (g/cm^3^)	Young’s Modulus (GPa)	Acoustic Impedance (10^5^ g/cm^2^ s)	Vickers Hardness (GPa)	Resistance to Brittle Fracturing (MPa m^1/2^)
Test method	PN-EN 993-1:1998	ASTM C 1419-99a		PN-EN-ISO 6507-1:2007	
Al_2_O_3_ with a 3.5 mm thickness	3.81 ± 0.1	472.6 ± 10.0	40.0 ± 0.2	18.9 ± 0.3	4.32 ± 0.3

**Table 4:** Validity of the groups of soft and hard ballistic insert properties being the criteria for selection of the optimal insert variants.

Property Groups	Feature	Validity (t_i_) ^1^
Physical properties	Areal density	3
	Thickness	2
Functional properties	V50 ballistic limit	3
	Estimated cost of materials for the ballistic inserts	1

^1^ Validity: from 1 (the least important feature) to 3 (the most important feature).

**Table 5:** Parameters of the designed para-aramid soft ballistic inserts assigned to two groups: physical and functional properties.

Sample	Physical Property Group		Functional Property Group	
	Areal Density	Thickness	V50 Ballistic Limit	Estimated Cost
-	kg/m^2^	mm	m/s	EUR
Twaron^®^ CT612 WRT soft ballistic insert				
CT612_1	2.5	3.6	438	9.45
CT612_2	4.5	6.5	585	17.10
CT612_3	5.0	7.1	620	18.90
CT612_4	6.1	8.2	645	22.50
CT612_5	7.5	10.0	700	28.35
Twaron^®^ UD42 soft ballistic insert				
UD42_1	1.9	2.2	368	4.80
UD42_2	3.1	3.5	455	7.80
UD42_3	3.6	4.1	495	9.00
UD42_4	5.0	5.7	540	12.60
UD42_5	7.0	6.8	600	15.00

**Table 6:** Selection of the optimal variants of para-aramid soft ballistic inserts.

Sample	Physical Properties Group		Functional Properties Group		General Coefficient of Quality (GSQ)	General Quality Class (GQC)
	SCQ_Ph_ ^1^ Factor	Quality Class (C_Ph_) ^2^ Factor	SCQ_U_ ^1^ Factor	Quality Class (C_U_) ^2^ Factor		
Twaron^®^ CT612 WRT soft ballistic insert						
CT612_1	0.86	1	0.36	6	0.61	4
CT612_2	0.50	5	0.61	4	0.55	4
CT612_3	0.41	6	0.67	3	0.54	5
CT612_4	0.24	8	0.69	3	0.47	5
CT612_5	0.00	10	0.75	2	0.38	6
Twaron^®^ UD42 soft ballistic insert						
UD42_1	1.00	0	0.25	7	0.63	4
UD42_2	0.38	6	0.41	6	0.38	6
UD42_3	0.72	3	0.49	5	0.61	4
UD42_4	0.49	5	0.56	4	0.52	5
UD42_5	0.22	8	0.67	3	0.43	6

^1^ The SCQs of the variants ranged from 0 to 1, where 1 stands for perfect quality. ^2^ C = 0 (ideal variant) and C = 9 (most unfavorable).

**Table 7:** Parameters of the designed para-aramid hard ballistic inserts selected in two groups: physical and functional properties.

Sample	Physical Properties Group		Functional Properties Group	
	Areal Density	Thickness	V50 Ballistic Limit	Estimated Cost
-	kg/m^2^	mm	m/s	EUR
Composite type 1—hard ballistic insert				
C1_1	9.2	3.0	698	7.50
C1_2	11.2	4.5	735	15.00
C1_3	15.0	8.0	984	30.00
C1_4	16.6	9.0	1015	34.50
C1_5	18.6	10.0	1042	37.50
C1_6	19.4	11.5	1095	45.00
C1_7	21.4	12.5	1084	52.50
Composite type 2—hard ballistic insert				
C2_1	23.0	7.0	1408	33.50
C2_2	27.0	11.5	1736	48.50
C2_3	29.0	12.5	1798	56.00
C2_4	30.6	13.3	1852	60.50
C2_5	31.5	14.5	1775	63.50
C2_6	34.2	16.5	1850	71.50

**Table 8:** Selection of the optimal variants of the para-aramid hard ballistic inserts.

Sample	Physical Properties Group		Functional Properties Group		General Coefficient of Quality (GSQ)	General Quality Class (GQC)
	SCQ_Ph_ ^1^ Factor	Quality Class (C_Ph_) ^2^ Factor	SCQ_U_^1^ Factor	Quality Class (C_U_) ^2^ Factor		
Composite type 1—hard ballistic insert						
C1_1	1.00	0	0.25	7	0.63	4
C1_2	0.84	2	0.28	7	0.56	4
C1_3	0.50	5	0.67	3	0.58	4
C1_4	0.38	6	0.70	3	0.54	5
C1_5	0.24	8	0.73	3	0.49	5
C1_6	0.14	9	0.79	2	0.47	5
C1_7	0.00	10	0.73	3	0.36	6
Composite type 2—hard ballistic insert						
C2_1	1.00	0	0.25	7	0.63	4
C2_2	0.6	4	0.70	3	0.65	3
C2_3	0.45	6	0.76	2	0.60	4
C2_4	0.33	7	0.82	2	0.57	4
C2_5	0.23	8	0.67	3	0.45	6
C2_6	0.00	10	0.75	3	0.37	6

^1^ The SCQs of the variants ranged from 0 to 1, where 1 stands for perfect quality. ^2^ GQC = 0 (ideal variant) and C = 9 (most unfavorable).

## Data Availability

Not applicable.
